# The relationship between medication literacy and skin adverse reactions in non-small-cell lung cancer patients undergoing targeted EGFR-TKI therapy

**DOI:** 10.1186/s12885-022-09599-w

**Published:** 2022-05-03

**Authors:** Ruofei Du, Huashan Yang, Huiyue Zhou, Lixia Ma, Mikiyas Amare Getu, Changying Chen, Tao Wang

**Affiliations:** 1 The College of Nursing and Health of Zhengzhou University, Zhengzhou, 450001 China; 2 Department of Quality Control, The First Affiliated Hospital of Zhengzhou University, Zhengzhou, 450052 China; 3 Academy of Medical Sciences of Zhengzhou University, Zhengzhou, 450001 China; 4 School of Statistics, Henan University of Economics and Law, Zhengzhou, 450046 China; 5 Telethon Kids Institute, Perth, WA 6872 Australia; 6 Medical School, University of Western Australia, Perth, WA 6872 Australia; 7 People’s Hospital of Hebi, Hebi, 458010 China

**Keywords:** Medication literacy, Skin adverse drug reactions, NSCLC, EGFR-TKI, Targeted therapy

## Abstract

**Background:**

High medication literacy is the basis of rational medication application and is essential for the management of severe adverse drug reactions. The objective of the present study was to assess the level of medication literacy and determine the association between medication literacy and skin adverse drug reactions in non-small-cell lung cancer (NSCLC) patients undergoing targeted epidermal growth factor receptor tyrosine kinase inhibitor (EGFR-TKI) therapy.

**Methods:**

This is a cross-sectional study conducted from May to September 2020. In total, 296 NSCLC patients undergoing targeted EGFR-TKI therapy were recruited from hospitals in Henan, China. Structured questionnaires were used to evaluate skin adverse drug reactions and medication literacy. Pearson correlation analysis and binary logistic regression analysis were carried out to identify the correlations between medication literacy and the severity of skin adverse drug reactions in the recruited patients.

**Results:**

The research sample consisted of 296 patients with a response rate of 92.5%. The mean score of skin adverse drug reactions and the mean score of medication literacy were 1.83 ± 0.91 and 6.54 ± 2.78, respectively. In total, 188 patients (63.5%) were considered to have moderate medication literacy. According to the binary logistic regression analysis, the following factors were associated with severe skin adverse drug reactions: age (B =  − 3.929, *P* = 0.000), sex (B = -4.062, *P* = 0.000), educational level (B = 2.712, *P* = 0.002), comorbidity (B = 3.297, *P* = 0.001), eczema history (B = 2.996, *P* = 0.001), nutritional status (B = -4.891, *P* = 0.000), blood interleukin-6 level (B = -2.143, *P* = 0.013), blood high-sensitivity C-reactive protein level (B = -4.015, *P* = 0.000), combination of drugs (B = -3.183, *P* = 0.048) and medication literacy (B =  − 1.503, *P* = 0.000). Subgroup analysis showed that in addition to medication literacy, some other factors including education level, comorbidity, nutritional status, blood interleukin-6 level and combined drug application were common factors that contributed to various adverse skin drug reactions in NSCLC patients under targeted EGFR-TKI therapy.

**Conclusion:**

The low medication literacy of the investigated NSCLC patients undergoing targeted EGFR-TKI therapy was correlated with a high proportion of severe skin adverse drug reactions. In addition, factors other than medication literacy including education level, comorbidity, nutritional status, blood interleukin-6 level and the combinatorial application of drugs were also related to the severity of various adverse skin drug reactions. A comprehensive and targeted intervention may be beneficial to improve medication literacy and control severe skin adverse drug reactions in NSCLC patients.

## Introduction

Globally, there were 2.22 million new lung cancer cases and 1.55 million lung cancer-related deaths in 2019, while the corresponding data reached 787,000 and 631,000 in China alone in 2015 [1, 2]. Among various types of lung cancers, non-small-cell lung cancer (NSCLC) accounts for 85% of the total cases in China [3]. Significantly, 75% of NSCLC patients are in advanced stages when diagnosed with a 5-year survival rate of approximately 15% [4]. As a result, NSCLC-related theranostics and clinical care have attracted significant attention in recent years.

The rapid development of molecular biology has enabled the identification of molecular targets of particular cancer cells, thus providing a framework for targeted anticancer therapy [5]. Several studies had shown that targeted therapies could improve the overall survival (OS), progression-free survival (PFS), and response rate (RR) of cancer patients and contributed to better tolerance and quality of life (QoL) [6]. The National Comprehensive Cancer Network (NCCN) guidelines confirmed that epidermal growth factor receptor tyrosine kinase inhibitors (EGFR-TKIs) could be used as the standard first-line treatment for EGFR mutation-positive NSCLC patients [7], which represent more than 50% of NSCLC patients in the Asia–Pacific region [8]. Partly because of its oral administration, EGFR-TKIs have gained increasing popularity in recent years [9], highlighting the importance of studying this group of drugs in clinical settings. For this purpose, EGFR-positive NSCLC patients who received EGFR-TKI treatments were investigated in this study. Importantly, although there are various types of EGFR-TKIs clinically available, they have very similar mechanisms of reaction [10], which enabled the comparison and analysis of the data collected from patients using different types of EGFR-TKIs.

Although the incidence of lethal adverse drug reactions (ADRs) of targeted therapy is lower than that of chemotherapy, due to the prolonged treatment time, an up to 80% ADR incidence has been reported [11]. Skin ADRs are the most common ADRs for EGFR-TKI therapy. As recorded by a recent investigation, approximately 80% of patients who received EGFR-TKI treatment showed skin ADRs within three months [12]. Severe skin ADRs can harm patients both physically and psychologically [13]. For example, the symptoms caused by skin ADRs could disturb patients’ sleep, detrimentally affect their self-esteem, and result in various social participation disorders [14, 15]. Even worse, it was reported that the medication adherence of patients receiving EGFR-TKI-based targeted therapy was poor, with 32% of NSCLC patients terminating treatments due to skin toxicity reactions, which compromised the treatment effects [16, 17].

Medication literacy refers to the degree to which individuals can obtain, understand, communicate, calculate and process specific information about their medications for informed decisions [18]. In previous studies, it was confirmed that patients with insufficient medication literacy showed poor medication practice (e.g. inappropriate self-medication behavior) and self-management, which is related to higher probability of hospitalization, a higher ADR rate and increased admission to the ICU [19–21]. Therefore, medication literacy is crucial for safe and effective therapeutics [22].

While EGFR-TKIs have emerged as a mainstream treatment for NSCLC, to the best of our knowledge, a systematic investigation of the impact of the status of medication literacy on skin ADRs has not been performed in this group of patients. However, this topic is of obvious importance considering that patients generally show less knowledge of this relatively new anticancer treatment scheme.

This work, therefore, provides valuable information about the relationship between the level of medication literacy and the severity of skin ADRs in NSCLC patients under EGFR-TKI treatment and facilitates rational drug management.

## Materials and methods

### Aim

This study aimed to assess the level of medication literacy of NSCLC patients undergoing targeted FGFR-TKI therapy and determine the association between the level of medication literacy and the severity of skin ADRs. Based on this knowledge, we endeavored to develop pertinent promotion strategies to manage skin ADRs for improved treatment outcomes.

### Design and setting

This work employed a cross-sectional study from May to September 2020. NSCLC inpatients and outpatients undergoing EGFR-TKI treatment were recruited from three tertiary hospitals in Henan, China.

In this study, medication literacy (Factor A) was a comprehensive index and an important predictor of rational medication that contained four aspects: medication knowledge, medication behavior, medication management skills and attitude towards medications [23]. Factor X was defined as the severity of skin ADRs. The hypotheses were: (1) NSCLC patients undergoing targeted EGRF-TKI therapy had low medication literacy; (2) NSCLC patients displayed better medication literacy (Factor A) tended to have mild skin ADRs (Factor X) after EGFR-TKI treatment.

### Participants

A total of 296 NSCLC patients undergoing EGFR-TKI treatment were recruited from hospitals in Henan, China. Convenience sampling method was used. The inclusion criteria for participation were as follows: (1) the pathological diagnosis was NSCLC; (2) EGFR-positive advanced NSCLC patients; (3) patients had been on EGFR-TKI treatment for at least four weeks; (4) patients only received EGFR-TKI targeted anticancer therapy; (5) patients were 18 ~ 70 years old; (6) patients could communicate normally; (7) patients were able to give informed consent. The exclusion criteria included: (1) patients were unaware of their disease conditions; (2) patients had difficulty completing the research due to poor physical function; and (3) patients were unable to complete the questionnaire either independently or with the assistance of investigators. Skin ADRs were identified based on the coexistence of the adverse symptoms upon EGFR-TKI treatment (the symptoms of skin ADRs appeared with the application of EGFR-TKI treatment and decreased or disappeared when EGFR-TKI treatment was reduced or stopped).

The sample size was calculated based on the following formula: $n=(\frac{1-\mathrm{\alpha }/2}{\updelta }$)$\times \mathrm{p}\times (1-\mathrm{p})$. After accounting for an attrition rate, the estimated minimum sample size was 186. Among 350 NSCLC patients with EGFR-TKI treatment, 333 met the inclusion criteria and were contacted. As 13 patients refused to participate and 24 patients did not complete all questionnaires, a total of 296 patients were included in this study.

### Procedure and data collection

The list of potential participants was obtained from the electronic medical records of the hospitals. After patient selection, the principal investigator (PI) explained information regarding the purpose, content, and investigation procedures as well as the principle of anonymity of the study to eligible participants. Upon agreement to participate in the study, informed consent forms were signed. Questionnaires were provided and completed by participants during hospitalization or clinical consultation.

After obtaining the consent of relevant departments, patients’ disease-related information was collected from medical records. Trained postgrad students were recruited to distribute questionnaires, respond to patients’ questions and assess the severity of skin ADRs. For participants who could not read and write, the interviews were completed with the assistance of researchers. The questionnaires were collected immediately after completion and checked for any missing information.

### Measures

#### Demographic characteristics

This was a self-designed questionnaire used to collect information on sociodemographic variables, such as gender, age, level of education, family monthly income, marital status, residence, type of medical insurance, disease stage, eczema history, nutritional status (determined by Patient-Generated Subjective Global Assessment (PG-SGA)), duration of treatment, combination of medicine and comorbidities (calculated as the number of chronic diseases included in the Charlson Comorbidity Index (CCI)). In addition, the effects of the blood interleukin-6 (IL-6) and the blood high-sensitivity C-reactive protein (hs-CRP) levels were also analysed. As most of the skin ADRs induced by targeted EGRF-TKI therapy occurred 1 ~ 3 months after treatment [24], these two factors were detected one month after treatment.

### Medication literacy scale adapted for the Chinese population

The medication literacy scale was originally developed by Sauceda et al. at the University of Texas in the United States in 2012 [25]. This tool was developed to assist pharmacists in evaluating and improving the medication literacy of patients. This scale was translated and adapted into the Chinese version in 2016 by Zheng Feng et al. [26]. The Chinese version of the medication literacy scale was developed for testing the medication literacy of the general population. However, the investigated participants in this study were patients with a specific disease (NSCLC patients undergoing targeted EGFR-TKI therapy). Therefore, we adjusted the contents of the scale to fit the population under study. We conducted two rounds of expert consultations to ensure the validity of the scale (see Table 2). Then, a presurvey was conducted before the formal survey to test the reliability and validity of the new scale. The scale assessed the patients' ability to extract and process discontinuous drug information (e.g., prescriptions, drug labels), which contained 14 items with a dichotomous scoring system (correct, 1; incorrect, 0). The total score of this scale is 14. The level of medication literacy fell into three groups according to the score: adequate literacy (> 10), marginal literacy (4 ~ 10), and inadequate literacy (< 4). That is, the higher the score is, the higher the level of medication literacy. The Cronbach's α was 0.77 ~ 0.89, and the content validity was 0.89 in this study. Compared with the commonly used health literacy assessment tools, an advantage of this tool is the reduced requirements of reading comprehension of patients.

### Common terminology criteria for adverse events version 5.0 (CTCAE 5.0)

The classification of skin ADRs in the latest edition of CTCAE 5.0 (the skin and subcutaneous tissue part) has been widely used in clinical practice [27]. CTCAE 5.0 described the specific definition, performance and nature of each skin ADR and divided ADRs into five grades according to the scope of occurrence, symptoms, impact on life (whether the ADR is life-threatening). According to the occurrence and the degree of ADR, skin ADRs were rated on a scale from 0 to 6 (0 indicated no skin ADR and 5 indicated the highest severity of skin ADR). According to the impact of ADR on life and daily activities, ≤ 2 points was defined as non-severe ADR, > 2 points was defined as severe ADR. In cases when the patients had multiple skin ADRs during targeted EGFR-TKI therapy, only the most serious ADRs were scored.

### Statistical analysis

Data were analysed by SPSS 21.0. Quantitative data were analysed using descriptive statistics to describe sample demographics and clinical variables. In the descriptive analyses, means and standard deviations were calculated for continuous data, while frequencies and percentages were computed for categorical variables. Pearson correlation analysis was applied to determine the correlations between medication literacy and the severity of skin ADRs.

After confirming the eligibility of the assumptions for logistic regression, binary logistic regression was performed to explore factors independently related to the severity of skin ADRs. The variables included in the logistic regression, such as comorbidity [28], eczema history [29], nutritional status [30], IL-6 [31], hs-CRP [32], duration of medicine [33], and combination of medicine [34] were recommended by previous studies. We also referred to the suggestions of clinicians. All statistical tests used were two-tailed, and *P*-value < 0.05 were considered statistically significant.

### Ethics approval and consent to participate

This study was approved by the Ethics Committee (No.ZZUIRB-2020–97) in the College of Nursing and Health of Zhengzhou University, and administrative permissions were obtained from directors of the Department of Oncology. All methods were carried out in accordance with relevant guidelines and regulations. Information about the study was presented to the participants and written informed consent was collected before the study.

## Results

### Demographics characteristics

In this study, a total of 320 questionnaires were distributed, of which 296 were completed, yielding a response rate of 92.5%. A total of 296 participants were included in the study with more men (54.4%) than women (45.6%). The ages of these patients ranged from 43 to 86 years. The mean age of the respondents was 66.9 years (SD = 9.78 years), with 71.6% over 60 years old. Other key characteristics of the sample were also included, as shown in Table 1.

**Table 1:** Patient characteristics (*n* = 296)

**Variables**		**Frequency(%)**
**Marital status**	With spouse	250 (84.5)
	Without spouse	46 (15.5)
**Education Level**	Junior school and below	94 (31.8)
	High school	158 (53.4)
	College and above	44 (14.8)
**Occupation status**	Employed	30 (10.1)
	Unemployed	266 (89.9)
**Residence**	Urban	114 (38.5)
	Rural	182 (61.5)
**Medical insurance**	Yes	231 (78)
	No	43 (22)
**Family income Chinese RMB (¥)/M/P**	< 1000/M	15 (5.1)
	1001 ~ 3000/M	113 (38.2)
	3001 ~ 5000/M	133 (44.9)
	> 5001/M	35 (11.8)
**CCI**	** > **2	178 (60.1)
	≤ 2	118 (39.9)
**Stages of disease**	Stage III	119 (40.2)
	Stage IV	177 (59.8)
**Eczema history**	Yes	40 (13.5)
	No	256 (86.5)
**Nutritional status**	Good	150 (50.7)
	Moderate malnutrition	114 (38.5)
	Severe malnutrition	32 (10.8)
**IL-6**	Normal	108 (36.5)
	Up	188 (63.5)
**hs-CRP**	Normal	105 (35.5)
	Up	191 (64.5)
**Duration of medicine (month,M)**	≤ 3	182 (61.5)
	> 3	114 (38.5)
**Combination of medicine**	Yes	202 (68.2)
	No	94 (31.8)

### Medication literacy of NSCLC patients with targeted EGRF-TKI therapy

Details of the medication literacy of the 296 investigated NSCLC patients are shown in Table 2. The mean score of medication literacy was 6.54 ± 2.78, which represented marginal literacy (4 ~ 10). The lowest and highest scores were 3 and 13, respectively. Forty-two (14.2%) patients obtained a score of > 10, which was considered as having "adequate medication literacy"; a significant portion of the participants 188 (63.5%) obtained a score of 4 ~ 10, which was considered as "marginal medication literacy", whereas 66 (22.3%) participants obtained a score of < 4, which was considered as "inadequate medication literacy".

**Table 2:** Medication literacy for NSCLC patients with targeted therapy (*n* = 296)

Items	Correct answers (%)
**Case scenario 1**	
A1:According to the labeled instruction, how many times per day should you take the medicine?	202 (68.2)
A2:Please tell me how much medicine you should take each time and take out the corresponding dose	129 (43.7)
A3:According to the instruction, please tell us or point out What are the adverse reactions of the medicine?	118 (39.8)
A4:According to the instruction, please tell me what do you need to pay attention to about the medicine?	120 (40.6)
A5:Looking at the prescription, if your medicine is run out, from whom you should get a new prescription?	152 (51.5)
**Case scenario 2**	
A6:Looking at the instructions on this package, how much dosing of the medicine should you take each time?	140 (47.3)
A7:If you know the dosage of the medicine that you need to take, please tell the total dosage and pour medicine you should take a day	122 (41.5)
A8:According to the directions, what is the maximum dosage should you take?	109 (36.9)
**Case Scenario 3**	
A9: Looking at this prescription, what is the name of the medicine that you need to buy at the pharmacy?	210 (71.1)
A10:According to the prescription, how do you store medicine you take?	135 (45.7)
A11:Looking at this bottle, the medicine in the bottle has the similar purpose with the medicine on the prescription, if you need to take 30 pills to treat the disease, how many boxes should you buy to make the correct amount of drug required by the original prescription?	179 (60.4)
**Case Scenario 4**	
A12: Looking at the box, when will the medicine expire?	200 (67.5)
A13:According to the directions, what is or what are the active ingredients in each pill?	124 (41.9)
A14: Please check the package carefully, in which cases should you stop the medicine?	129 (43.6)

### Skin ADRs of NSCLC patients with targeted EGFR-TKI therapy

The ADR scores ranged from 0 to 5 on the CTCAE 5.0. The mean score for skin ADRs was 1.83 (SD = 0.91). Based on the CTCAE 5.0 score, patients were categorized into two groups (non-severe or severe ADR) according to whether skin ADRs affected daily activities. The results indicated that 118 respondents (39.9%) had severe skin ADRs and 178 (60.1%) had non-severe **s**kin ADRs. Participants may have more than one skin ADR, the type and degree of each skin ADR are shown in Table 3. Detailed information of different phenotypes on the EGFR-TKI-induced skin ADRs is shown in Table 4.

**Table 3:** Skin ADRs of NSCLC patients with targeted therapy

ADR score	Rash (n)	Hand-foot syndrome (n)	Xerosis (n)	Pruritus (n)	Nail change (n)	Nail loss (n)	Hair loss (n)	Total (n)
1	60	52	41	24	27	14	23	241
2	73	51	48	29	18	9	29	257
3	60	55	34	26	0	0	0	175
4	26	0	0	0	0	0	0	26
5	3	0	0	0	0	0	0	3
**Total (n)**	222	158	123	79	45	23	52	702

**Table 4:** Characteristics of different skin ADRs

Skin ADRs	Mean score (x ± s)	Incidence (%)	≥ 3 Level Incidence (%)	Manifestation	Average time of occurrence	Location	Concomitant symptoms
Rash	2.27 ± 1.68	75	30.1	Papule; Macula; Pustule	21 days	Face (87%); Back and chest (69%); Lower limbs (38%); Abdomen (26%)	Itch; Pain; Skin burning; Skin tightness
Hand-foot syndrome	2.02 ± 0.82	53.4	18.6	Skin redness; Pain; Swelling; Numbness	32 days	Palms and soles	Increased skin sensitivity; Skin peeling, blisters, bleeding, chapping, and excessive keratosis
Xerosis	1.94 ± 0.79	41.6	11.5	The skin becomes thin, shriveled, blackened, unresponsive; Skin wrinkles and skin aging	42 days	Limbs	Skin cracks; Pain; Itch; Blooding; Desquamation
Pruritus	2.02 ± 1.40	26.7	8.9	Itch; Dry	53 days	Systemic or local	Edema; Papules; Lichenification; Sclerosis, Exudation; Crusting
Nail change	1.40 ± 0.49	15.2	0	Granuloma around nails	49 days	Nails and tissues around nails	Pain; Suppuration; Erythema; Swelling; Lateral nail fissure
Nail loss	1.39 ± 0.49	7.8	0	The nail bed is separated from the deck	38 days	Nails	Paronychia; Infection; Nail falling off; Nail lesions
Hair loss	1.56 ± 0.50	17.6	0	Hair loss; Hair curly, thin and fragile	33 days	Head; Eye; Face	Eyelashes are thick, long and curly; Facial hairiness

### Correlations between NSCLC patients' medication literacy and the severity of skin ADRs

Pearson correlation analysis of the main variables indicated that medication literacy was negatively correlated with skin ADRs (*r* = -0.691, *P* = 0.000). We also analysed the correlations between medication literacy and various types of skin ADRs in detail (Table 5).

**Table 5:** Correlations between medication literacy and the severity of skin ADRs

	**Medication literacy**	***p***
Skin ADRs	-0.691	0.000
Rash	-0.744	0.000
Hand-foot syndrome	-0.539	0.000
Xerosis	-0.486	0.005
Pruritus	-0.577	0.000
Nail change	-0.012	0.834
Nail loss	-0.032	0.542
Hair loss	-0.027	0.644

*P* values < 0.01 were considered statistically significant (two-tailed)

### Factors associated with the severity of skin ADR prediction

Binary logistic regression analysis was performed to examine skin ADR-related factors. All variables, including demographic characteristics and medication literacy were entered by stepwise variable selection with the forward selection and backwards elimination methods combined to filter the independent variables. For effective analysis, patients were divided into two groups (non-severe and severe skin ADRs) based on CTCAE 5.0 scores. As shown in Table 6, the binary logistic regression analysis identified significant factors for the prediction of the severity of skin ADRs. The model could explain 68.9% of the change in skin ADR levels. Significant factors that were independently associated with the severity of skin ADRs were as follows: age [OR 0.020 (95% CI: 0.002–0.160); *P* = 0.000], gender [OR 0.017 (95% CI: 0.002–0.129); *P* = 0.000], education level [OR 0.066 (95% CI: 0.012–0.373); *P*  = 0.002], eczema history [OR 0.073 (95% CI: 0.016–0.826); *P*  = 0.000], nutritional status [OR 0.039 (95% CI: 0.125–0.769); *P*  = 0.001], the blood IL-6 level [OR 0.362 (95% CI: 0.188–0.833); *P*  = 0.013], the blood hs-CRP level [OR 0.407 (95% CI: 0.009–0.552); *P*  = 0.000], combination of medicine [OR 0.041 (95% CI: 0.002–0.975); *P*  = 0.048], CCI [OR 0.037 (95% CI: 0.006–0.243); *P*  = 0.001] and medication literacy [OR 0.223 (95% CI: 0.112–0.373); *P*  = 0.000]. Individuals with comorbidities, combined drug application, eczema history, malnutrition, elevated blood IL-6 and hs-CRP levels, older age, lower education level, worse medication literacy were more likely to have severe skin ADRs. Interestingly, the sex of male was also identified as a factor for severe skin ADRs. Table 7 shows the independent variable assignment of binary logistic regression analysis of NSCLC patients' skin ADRs.

**Table 6:** Binary logistic regression analysis for factors predicting the severity of skin ADRs

Variables	severe skin ADR/non-severe skin ADR			
	β	OR	95%CI	*p*-value
Age	-3.929	0.020	0.002–0.160	0.000
Gender	-4.062	0.017	0.002–0.129	0.000
Marital status	0.366	1.442	0.119–17.498	0.774
Education level	-2.712	0.066	0.012–0.373	0.002
Occupation status	-0.535	0.586	0.101–3.387	0.550
Residence	-1.517	0.219	0.039–1.235	0.085
Medical insurance	-1.032	0.356	0.043–2.929	0.337
Family income Chinese RMB (¥)/M/P	-0.445	0.641	0.245–1.673	0.363
CCI	-3.297	0.037	0.006–0.243	0.001
Disease stage	-3.578	2.023	0.769–13.339	0.732
Eczema history	-2.996	0.073	0.016–0.826	0.001
Nutritional status	4.891	0.039	0.025–0.769	0.000
Blood IL-6 level	2.143	0.362	0.188–0.633	0.013
Blood hs-CRP level	4.015	0.047	0.019–0.552	0.000
Duration of medicine (month,M)	0.172	1.188	0.216–6.633	0.843
Combination of medicine	-3.183	0.041	0.002–0.975	0.048
Medication literacy	-1.503	0.223	0.112–0.373	0.000

F = 213.74; R2 = 69.6%, adjusted R2 = 68.9%

Binary logistic regression model was computed for each outcome separately; the severity of skin ADRs was included as an independent variable, each model was adjusted by age, gender, marital status, education level, residence, occupation status, medical insurance, family income, CCI, disease stage, eczema history, blood IL-6 level, blood hs-CRP level, duration of medicine and combinatorial application of drugs

**Table 7:** Independent variables assignment of binary logistic regression. Analysis of NSCLC patients' skin ADRs

Independent Variables	Assignment
Age	≥ 60 = 1; < 60 = 2
Gender	male = 1; female = 2
Marital status	with spouse = 1; without spouse = 2
Education level	junior school or below = 1; high school = 2; college or above = 3
Occupation status	employed = 1; unemployed = 2
Residence	urban = 1; rural = 2
Medical insurance	yes = 1; no = 2
Family income Chinese RMB (¥)/M/P	< 1000 = 1; 1001 ~ 3000 = 2; 3001 ~ 5000 = 3; > 5001 = 4
CCI	> 2 = 1; ≤ 2 = 2
Disease stage	stage I = 1; stage II = 2; stage III = 3; stage IV = 4
Eczema history	yes = 1; no = 2
Nutritional status	good = 1; moderate = 2; sever = 3
Blood IL-6 level	normal = 1; up = 2
Blood hs-CRP level	normal = 1; up = 2
Duration of medicine (month,M)	≤ 3 = 1; > 3 = 2
Combination of medicine	yes = 1; no = 2
Medication literacy	Continuous value

### Subgroup analysis

The skin ADRs related to targeted EGFR-TKI therapy are diversified. A further subgroup analysis of factors for the severity of different phenotypes of skin ADRs was necessary. After stratification, due to the small sample sizes, nail changes, nail loss and hair loss were not analysed to avoid bias. Meanwhile, among the patients with xerosis and pruritus, the proportion of patients with severe ADRs was small, which led to a reduction in the accuracy of the analysis. As a result, these ADRs were not analysed. The binary logistic regression analysis identified significant factors for the severity of rash and hand-foot syndrome, as shown in Tables 8 - Table  9. Individuals with comorbidities, combination of medicine, eczema history, malnutrition, elevated blood IL-6 and hs-CRP, older age, lower education level, longer medication and lower medication literacy were more likely to suffer severe rash. Individuals with comorbidities, a combination of medicine, malnutrition, elevated blood IL-6, lower education level, and lower medication literacy were more likely to have severe HFS. The independent variable assignments of binary logistic regression analysis were the same as those in Table 7.

**Table 8:** Binary logistic regression analysis for factors predicting the severity of rash

Variables	severe rash/non-severe rash			
	β	OR	95%CI	*p*-value
Age	-4.525	0.011	0.000–0.409	0.015
Gender	-2.112	0.121	0.010–1.479	0.121
Marital status	-0.137	0.842	0.105–7.532	0.827
Education level	-2.767	0.069	0.087–0.971	0.021
Occupation status	-1.535	0.659	0.001–21.623	0.325
Residence	-0.169	0.884	0.183–7.727	0.836
Medical insurance	1.537	6.322	0.678–29.683	0.092
Family income Chinese RMB (¥)/M/P	-0.469	0.647	0.175–2.531	0.529
CCI	-3.166	0.048	0.002–0.539	0.012
Disease stage	-0.579	0.654	0.866–19.231	0.507
Eczema history	-4.156	0.027	0.006–0.739	0.001
Nutritional status	2.976	8.870	2.755–17.046	0.004
Blood IL-6 level	3.177	3.116	1.892–19.385	0.014
Blood hs-CRP level	1.410	4.098	2.868–18.194	0.009
Duration of medicine (month,M)	-2.768	0.088	0.016–0.670	0.026
Combination of medicine	-4.151	0.061	0.001–0.359	0.004
Medication literacy	-1.594	0.203	0.069–0.595	0.004

F = 296.35; *R*^2^ = 72.5%, adjusted *R*^2^ = 71.1%

## Discussion

In this cross-sectional study, we started by analyzing the status of skin ADRs (e.g. the manifestations, time of occurrence, location, scope and severity) in NSCLC patients undergoing targeted EGFR-TKI therapy. It was quite clear that skin ADRs were a concern for EGFR-TKI therapy. Skin ADRs such as rash, xerosis, skin pruritus, hand-foot syndrome, paronychia, nail loss and hair loss were mostly recorded, similar to some published studies [35, 36].

**Table 9:** Binary logistic regression analysis for factors predicting the severity of hand-foot syndrome

Variables	severe hand-foot syndrome/non-severe hand-foot syndrome			
	β	OR	95%CI	*p*-value
Age	-2.339	0.396	0.025–1.370	0.396
Gender	-3.541	0.228	0.033–3.412	0.239
Marital status	-0.278	0.896	0.275–3.331	0.719
Education level	-0.799	0.544	0.196–0.819	0.028
Occupation status	-1.833	0.776	0.023–17.107	0.457
Residence	-0.335	0.678	0.111–9.975	0.866
Medical insurance	2.043	7.912	0.735–16.566	0.184
Family income Chinese RMB (¥)/M/P	-0.528	0.774	0.095–3.265	0.668
CCI	-1.335	0.424	0.112–0.886	0.025
Disease stage	-0.885	0.715	0.237–9.649	0.175
Eczema history	-0.480	0.828	0.054–1.956	0.728
Nutritional status	0.927	2.176	1.685–6.073	0.017
Blood IL-6 level	1.370	5.354	1.537–11.818	0.005
Blood hs-CRP level	0.410	3.192	0.476–8.748	0.509
Duration of medicine (month,M)	-0.879	0.417	0.177–2.092	0.067
Combination of medicine	-1.549	0.187	0.080–0.564	0.002
Medication literacy	-0.566	0.612	0.334–0.806	0.015

F = 184.55; *R*^2^ = 65.4%, adjusted *R*^2^ = 63.2%

Currently, there is no consensus about the incidence of severe skin ADRs. For example, a 24% severe skin ADR rate was reported by Sano and colleagues, which is 1.5 times lower than the current investigation (39.9%) [37]. This difference may be because of the variation in the type of cancer and the type of drugs under investigation. In fact, compared with other targeted therapies (e.g. ALK-TKI and VEGFR), EFGR-TKI treatments result in more severe skin ADRs, which is especially true for lung cancer treatment, in which EGFR-TKIs have been commonly used. However, in another study, Braden recorded an up to 55% severe skin ADR rate for lung cancer patients treated with EGFR-TKIs [38]. Upon close investigation, this high incidence was probably caused by the selected patients. As shown in Braden’s study, only advanced cancer patients with poor nutritional status were included. As malnourished patients often have edema, dryness, and poor elasticity of the skin [39], and all of which contribute to severe skin ADRs [40], it may be necessary for health care providers to supply professional nutritional support (e.g. adjusting the gastrointestinal function and enhancing the metabolic capacity of patients) to improve their nutritional status.

Then, based on previous reports that patients with higher medication literacy tended to manage skin ADRs better [41], we investigated the status of medication literacy of NSCLC patients undergoing EGFR-TKI therapy and comprehensively analysed the association between medication literacy and skin ADRs in this group of patients. As demonstrated, the investigated NSCLC patients showed only marginal medication literacy for EGFR-TKIs. As expected, a negative correlation between medication literacy and skin ADRs was recorded: the higher the score of medication literacy (patients had higher medication literacy), the lower the score of skin ADRs (patients had a lower degree of skin ADRs). These results highlighted the importance of evaluating the medication literacy of NSCLC patients for efficient EGFR-TKI treatment.

Studies had shown that skin ADRs of targeted EGRF-TKI therapy could be controlled through rational drug administration [29, 42], which relied not only on the expertise of clinical pharmacists but also on the level of self-medication management of patients. The level of self-medication in turn is decided by the medication literacy of patients [43]. As demonstrated in this study, medication literacy was indeed a crucial indicator in medication self-management of NSCLC patients under EGFR-TKI treatment and should be closely monitored by medical staff [44].

The mean score for the medication literacy scale was recorded as 6.54 (SD = 2.78), which represented marginal literacy [4–10] in this study. Due to the lack of medication literacy assessment studies of cancer patients worldwide, it was not feasible to compare the medication literacy level of patients in a comparable population. Alternatively, we interpreted the medication literacy score by comparing it with the full score of 14. According to the specific cut-off point for high (> 10), medium (4 ~ 10), and low (< 4) levels of medication literacy, we found that the medication literacy level for NSCLC patients undergoing EGFR-TKI therapy was sub-optimal. This is consistent with a previous study where a mean medication literacy score of 7.49 was reported [45].

Although medication literacy showed negative correlations with the severity of ADRs such as rash, xerosis, hand-foot syndrome and pruritus, it seemed to show less correlations with the severity of some ADRs including nail change, nail loss and hair loss. This may be due to the insufficient sample size. In future research, more samples are required to confirm the correlations between medication literacy and the severity of different skin ADRs.

It is clear from this study that better medication literacy contributes to efficient skin ADR control in NSCLC patients [46]. A plausible explanation is that patients with higher medication literacy had a better understanding and could better deal with skin ADRs. For example, higher medication literacy allowed patients to identify early signs of severe skin ADRs and seek professional medical advice [47], which were essential for the safety, effectiveness, and completion of the targeted therapy. In contrast, patients with lower medication literacy were more likely to perform incorrect self-evaluation (more likely to become victims of drug abuse), leading to severe skin ADRs [48]. Therefore, to improve the overall treatment outcomes of NSCLC patients with targeted EGRF-TKI therapy, it is necessary to improve medication literacy through multiple strategies such as increasing patients' awareness of health education, improving communication with health care providers, promoting learning skills, and establishing multidisciplinary team management.

The current research revealed that the management of skin ADRs involved specialized knowledge in medicine, nursing, pharmacy, nutrition, and psychology. As a result, it may beneficial to establish an effective multidisciplinary team through the following strategies: (1) train nurses knowledge about targeted cancer therapy; (2) include pharmacists, nutritionists, and psychologists in the team and define the responsibility and performance standards of each member of the multidisciplinary team; and (3) develop and cultivate core competence. Furthermore, the next three strategies are also useful to improve medication literacy, including (1) using social resources and government support to strengthen public cognition; (2) initiating the screening of patients with lower medication literacy criteria to identify individualized health education; and (3) improving the availability and convenience of obtaining medical information through different types of new media (i.e., publications, audio recordings, and videos).

At the end of this study, to provide additional information, other potential influencing factors of skin ADRs were also investigated through subgroup analyses. It was disclosed that apart from medication literacy, quite a few factors such as education level, comorbidity, nutritional status, blood IL-6 level and combined drug application also significantly affect the severity of skin ADRs.

As a cross-sectional study, the patients were not followed up in the current work. In a recent clinical trial, it was confirmed that the severity of skin ADRs not only affected the treatment effects but was also positively correlated with patients’ RR and OS [49]. For this reason, it seems necessary to perform a longer follow-up to further study the relationships between skin ADRs and these key evaluation indicators of cancer treatment (e.g. RR & OS) in NSCLC patients under EGFR-TKI treatment. In addition, the impacts of skin ADRs on patients also include patients' QoL and physiological/ psychological statuses. Exploration of these indicators will be equally important to direct clinical practice. Moreover, as previously reported, EGFR-TKIs of different generations demonstrated very different ADR patterns. For example, the rash and paronychia incidence of afatinib, a second-generation EGFR-TKI, was recorded as high as 89.1% and 56.8% [50, 51], while the corresponding data the first-generation EGFR-TKI (gefitinib and erlotinib) was generally lower (rash was 66.2% ~ 73% and paronychia was 4%—13.5%) [52, 53]. The third-generation EGFR-TKI recorded the lowest rash incidence [54]. Taking this issue into account, in future studies, subgroup analyses should be performed to explore the skin ADRs of different types of EGFR-TKIs and their impacts on patients.

Targeted EGFR-TKI therapy could cause drug hypersensitivity. These hypersensitivity reactions are more serious or even fatal and are different from general skin ADRs [55]. First, the incidence of hypersensitivity reactions is rare, while the incidence of general skin ADR reaches 80% [56]. Second, EGFR-TKIs selectively inhibit the EGFR signal and result in EGFR-related skin ADRs [57], while the pathological mechanism of hypersensitivity may be associated with either epidermal differentiation/re-epithelialization or cytotoxic T-cell-mediated adaptive immune responses [58]. Last, the clinical manifestations are different. Compared with EGFR-related skin ADRs as discussed in this article, the skin symptoms caused by drug hypersensitivity include maculopapular exanthema, Stevens Johnson syndrome, toxic epidermal necrolysis, etc. [59]. As the skin toxicity caused by hypersensitivity reactions has little correlation with medication literacy [59], this type of ADR was not studied in the current work. During our study, no EGFR-TKI-associated hypersensitivity was recorded.

## Strengths and limitations

First, to our knowledge, this is the first study on the medication literacy of cancer patients in China. With the increased application of oral anticancer drugs, medication literacy is playing an increasing role in improving self-medication management of patients. While the concept of medication literacy was formally defined only recently (in 2017), this study has filled in the gap in the understanding of medication literacy of cancer patients; Second, previous studies had mainly focused on the mechanism and effect of targeted therapy, with few studies involving ADRs (especially skin ADRs), this work provided important reference in this area. Third, this study analysed controllable factors such as cognition, nutrition and medication literacy, which provided directions for the intervention and management of ADRs.

There are some limitations of this study. The skin ADRs of different types of EGFR-TKIs are different. However, this study did not discuss the specific impacts of different EGFR-TKIs on patients, which will be emphasized in future research. Correlation analysis and regression analysis cannot explain the causal conclusions. Therefore, experimental research should be designed to explore the causal relationships between medication literacy and skin ADR in the future. In addition, the associations between skin ADRs and treatment effects, compliance and prognosis of NSCLC patients need to be further explored through long-term follow-up studies. The tool used for assessing medication literacy was adjusted based on an original scale which was designed for general population(as shown in the method section, strategies were used to ensure the rationality of this tool in the current study). In the future, it is better to develop a medication literacy assessment tool specifically for cancer patients. Additionally, this study was performed in a single region, which may compromise the reproducibility of the results. Further studies including a larger sample and more areas should be carried out. Most of the participants (71.6%) were older persons (≥ 60 years); thus, we obtained limited information about medication literacy and skin ADRs for younger patients, to whom the conclusions of our study might not be generalized.

## Conclusion

Our findings indicate a significant association between education level, comorbidity, nutritional status, blood IL-6 level, the combination of drugs, medication literacy and various skin ADRs in NSCLC patients undergoing targeted EGFR-TKI therapy. The incidence of severe skin ADRs was high. The level of medication literacy among patients with targeted EGRF-TKI therapy is sub-optimal and needs to be improved. Our study provides preliminary information and recommendations for developing effective health care intervention programs.

## Data Availability

The data generated during and/or analysed during the current study are not publicly available but are available from the corresponding author who was an organizer of the study.

## Abbreviations

ADR: Adverse Drug Reactions
NSCLC: Non-Small-Cell Lung Cancer
OS: Overall Survival
PFS: Progression-Free Survival
RR: Response Rate
EGFR-TKI: Epidermal Growth Factor Receptor Tyrosine Kinase Inhibitors
PG-SGA: Patient-Generated Subjective Global Assessment
IL-6: Interleukin-6
hs-CRP: high-sensitivity C-Reactive Protein
CCI: Charlson Comorbidity Index
CTCAE5.0: Common Terminology Criteria for Adverse Events Version 5.0
